# Transarterial Chemoembolization for the Treatment of Advanced Hepatocellular Carcinoma with Portal Vein Tumor Thrombosis: Prognostic Factors in a Single-Center Study of 188 Patients

**DOI:** 10.1155/2014/194278

**Published:** 2014-04-08

**Authors:** Lei Liu, Cheng Zhang, Yan Zhao, Xingshun Qi, Hui Chen, Wei Bai, Chuangye He, Wengang Guo, Zhanxin Yin, Daiming Fan, Guohong Han

**Affiliations:** ^1^Department of Liver Diseases and Digestive Interventional Radiology, Xijing Hospital of Digestive Diseases, Xijing Hospital, Fourth Military Medical University, No. 15 West Changle Road, Xi'an 710032, China; ^2^State Key Laboratory of Cancer Biology and Xijing Hospital of Digestive Diseases, Xijing Hospital, Fourth Military Medical University, Xi'an 710032, China

## Abstract

Transarterial chemoembolization (TACE) could achieve a better survival benefit than conservative treatment for advanced hepatocellular carcinoma (HCC) with portal vein tumor thrombosis (PVTT). In this retrospective study, all HCC patients with Child-Pugh score <7 and PVTT who were consecutively admitted to our center between January 2006 and June 2012 and underwent TACE were enrolled. The efficacy and safety of TACE were analyzed. Prognostic factors were determined by Cox regression analysis. Of the 188 patients included, 89% had hepatitis B virus infection, 100% were at Barcelona Clinic Liver Cancer stage C, and 81% (*n* = 152) and 19% (*n* = 36) were at Child-Pugh classes A and B, respectively. The incidence of procedure-related complications was 88%. No procedure-related death was found. The median overall survival was 6.1 months. Type of PVTT (hazard ratio [HR] = 2.806), number of tumor lesions (HR = 2.288), Child-Pugh class (HR = 2.981), and presence of metastasis (HR = 1.909) were the independent predictors of survival. In conclusion, TACE could be selectively used for the treatment of advanced HCC with PVTT. But a high rate of postoperative adverse events should not be undermined in spite of no procedure-related death. Preoperative type of PVTT, number of tumor lesions, Child-Pugh class, and metastasis could predict the prognosis of these patients.

## 1. Introduction

Hepatocellular carcinoma (HCC) is the sixth most common malignancy and is the third leading cause of cancer-related mortality worldwide [1, 2]. Indeed, a large number of HCC patients are diagnosed at intermediate or advanced stage, in which curative therapy, such as hepatic resection, radiofrequency ablation, and liver transplantation, could not be performed [3, 4]. According to the Barcelona Clinic Liver Cancer (BCLC) group recommendations [3, 4], transarterial chemoembolization (TACE) should be the standard treatment option for intermediate HCC (BCLC B stage) [5].

Portal vein tumor thrombus (PVTT) is negatively associated with the HCC patients' survival [6]. And it is traditionally considered as the contraindication for TACE [4], because the presence of PVTT potentially induces the development of acute liver failure or infarction after TACE. For advanced HCC patients with PVTT, sorafenib monotherapy demonstrated significant survival benefits in two large multicenter randomized controlled trails [7, 8]. However, a retrospective study by Pinter et al. compared the efficacies of TACE and sorafenib in advanced stage HCC patients (35% of patients treated with TACE had PVTT) and found there was no significant difference between these two treatments in terms of overall survival. Notably, the median overall survival in TACE group was longer than that in sorafenib group (9.2 months versus 7.4 months) [9].

Furthermore, several studies have shown that TACE could be safely performed in HCC patients with PVTT and might improve the survival [10–12]. More recently, a meta-analysis of 8 comparative studies, including 3 prospective and 5 retrospective studies, further confirmed this survival benefit for advanced HCC with PVTT, even with main portal vein obstruction [13]. Herein, we conducted a large-sample retrospective study of 188 advanced patients with PVTT to analyze the safety and efficacy of TACE and to determine the prognostic factors.

## 2. Patients and Methods

All advanced HCC patients with PVTT treated with TACE in our center between January 2006 and June 2012 were enrolled in this retrospective study. The diagnosis of HCC was based on the recommendations of the European Association for the Study of Liver Disease (EASL) or American Association for the Study of Liver Diseases (AASLD) [3, 4]. Eligibility criteria were as follows: (1) Eastern Cooperative Oncology Group score 0–2 points; (2) Child-Pugh score 5–7 points; and (3) the absence of portal cavernoma that is characterized by a tangle of tortuous hepatopetal collateral veins bypassing the occluded portal vein for the patent segmental vessel. This retrospective study was approved by the institutional review board of Xijing Hospital. Before treatment, all patients received the detailed information about the TACE operation and provided the written informed consent for the TACE operation. Baseline data before TACE were extracted from the medical records. Patients underwent routine follow-up physical examinations, laboratory tests (blood count, liver function tests), and CT during weeks 4 and 8 after initiation of treatment and every 8 weeks thereafter. The chest X-ray was also performed every 8 weeks. The end of follow-up was either death or December 2012.

In our study the type of PVTT was divided as follows: Type I, tumor thrombosis involving the main portal vein trunk; Type II, tumor thrombosis involving right/left portal vein or segment branches without involving main portal vein trunk.

### 2.1. TACE Procedure

TACE procedure has been previously described [14]. TACE consisted of an injection containing a mixture of chemotherapeutic agents and lipiodol (2–20 mL) followed by embolization with gelatin foam or polyvinyl alcohol until complete stasis was achieved in the tumor-feeding vessels; the chemotherapeutic agents used concurrently included doxorubicin (10–50 mg), epirubicin (10–50 mg), cisplatin (10–110 mg), and/or mitomycin (2–10 mg). Before January 2011, a combination of doxorubicin with epirubicin, cisplatin, or mitomycin was used. After that, doxorubicin alone was used. Tumor-feeding vessels were selected/superselected whenever possible. TACE was repeated “on demand” depending on the radiological response. When residual viable tumors were confirmed or new lesions developed in patients with adequate hepatic function, repeated TACE procedures were carried out. TACE-related complications were carefully recorded. Postembolization syndrome included fever, nausea, vomiting, and abdominal pain [15].

### 2.2. Evaluation of the Effects of TACE

The routine follow-up program was uniform for all patients and included a serum AFP assay, abdominal ultrasonography, and liver function test every one month at the first year and every three months thereafter. Contrast-enhanced CT scans were performed every 1-2 months during the first 3 months to evaluate the tumor response. The Response Evaluation Criteria in Solid Tumors (RECIST) was used to measure the tumor response: CR (complete response) is disappearance of all target lesions; PR (partial response) is 30–99% decrease in the sum of the longest diameter of the target lesions; SD (stable disease) is neither PR nor progressive disease; PD (progressive disease) is more than 20% increase in the sum of the longest diameter of the target lesions. Thereafter, contrast-enhanced CT scan was performed every 3 months for surveillance.

### 2.3. Statistical Analysis

Continuous variables were summarized as the mean values (± standard errors); categorical variables were expressed as frequencies. Median overall survival was calculated according to the Kaplan-Meier method and was compared using the log-rank test. Overall survival was measured from the date of TACE procedure to death from any cause. Univariate and multivariate Cox regression analysis was performed to assess the prognostic factors related to patient's survival. Variables included in the univariate analysis were gender (female versus male), age, ascites (yes versus no), red blood cell count, white blood cell, hemoglobin concentration, prothrombin time, international normalized ratio, alanine aminotransferase level, aspartate aminotransferase level, alpha fetoprotein, albumin level, total bilirubin level, creatinine, ECOG score (0 versus 1-2), Child-Pugh class (class A versus class B), type of PVTT (Type I versus Type II), tumor size, number of tumor lesions (≥3 versus <3), bilobar (yes versus no), extrahepatic metastasis (yes versus no), and arteriovenous fistula (yes versus no). A hazard ratio (HR) with 95% confidence interval (CI) was calculated for each variable. All variables with *P* < 0.10 in univariate analyses were included in the subsequent multivariate analysis. *P* value of <0.05 was considered the level of significance. All statistical calculations were performed using SAS 9.3 (Statistical Analysis System, SAS Institute Inc., USA).

## 3. Results

During the enrollment period, a total of 296 advanced HCC patients with PVTT were admitted to our center. Among them, 108 patients were excluded from this study, because 19 patients presented with portal cavernoma and 89 patients had a Child-Pugh score >7 points. Thus, 188 patients were enrolled.

Baseline characteristics were summarized in Table 1. Hepatitis B virus infection was the most common etiology of HCC. All patients were classified as BCLC C stage. Among them, 152 and 36 patients were Child-Pugh classes A and B, respectively. 22 patients had extrahepatic metastasis, mainly in lung, abdominal lymph node, and bone. 90 patients had tumor thrombosis in main portal vein and 98 in portal vein branches. The number of tumor lesions was >3 and <3 in 101 and 86 patients, respectively. The mean number of TACE sessions was 1.87 (1–9). The majority of patients (150; 79.8%) had 1-2 sessions, while 27 (14.4%) had 3-4 sessions, 10 (5.3%) had 5-6 sessions, and 1 (1%) had 9 sessions. The mean follow-up time was 8.6 months (95% CI 1.7–29.8). Hospitalization duration was 7 days in 123 patients, 8 days in 14 patients, 9 days in 22 patients, 10 days in 18 patients, 11 days in 6 patients, and 12 days in 5 patients.

### 3.1. Safety

The incidence of procedure-related complications was 88%. The most common complication was abdominal pain (75%), fever (71.3%), abdominal distension (28.2%), nausea (26.1%), and fatigue (7.4%). 16 patients had no complication after TACE treatment, 17 had one type of complication, 108 had two types of complications, 34 had three types of complications, and 13 had four types of complications. Most of the TACE-related complications occurred 2 or 3 days after the procedure in hospital and lasted up to 10 days. All complications were safely controlled by conservative treatment. No procedure-related death was recorded.

### 3.2. Efficacy

After TACE, the assessment of tumor response using the RECIST criteria classified 0 (0%), 31 (26.3%), 116 (61.7%), and 41 (21.8%) patients as CR, PR, SD, and PD, respectively.

**Figure 1:**
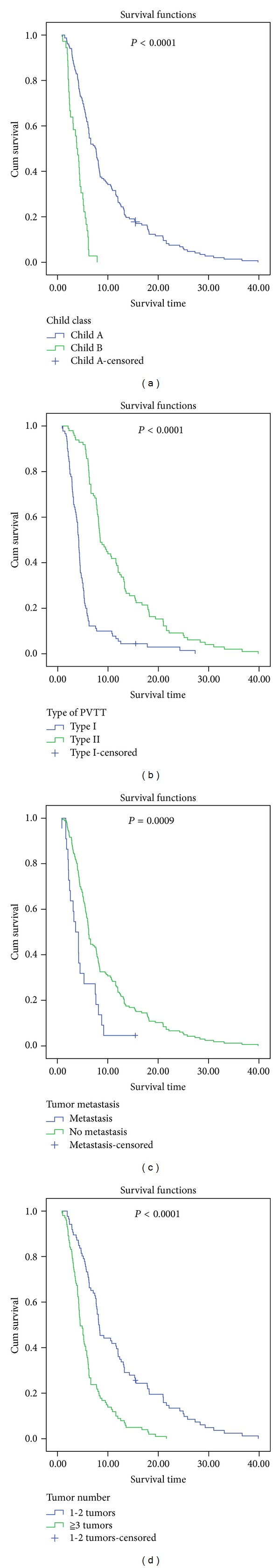
Comparison of overall survival in advanced hepatocellular carcinoma patients with portal vein tumor thrombosis according to the Child-Pugh classification (a), type of portal vein tumor thrombosis (b), metastasis (c), and number of tumor lesions (d).

By December 2012, only one patient had survived. The median overall survival was 6.1 months (95% CI: 5.6–6.5). Survival rates at 1 year and 2 years were 21.3% and 5.5%, respectively. The median survival time was significantly longer in Child-Pugh A patients than in Child-Pugh B patients (7.5 months versus 3.8 months, *P* < 0.0001) (Figure 1(a)).

**Figure 2:**
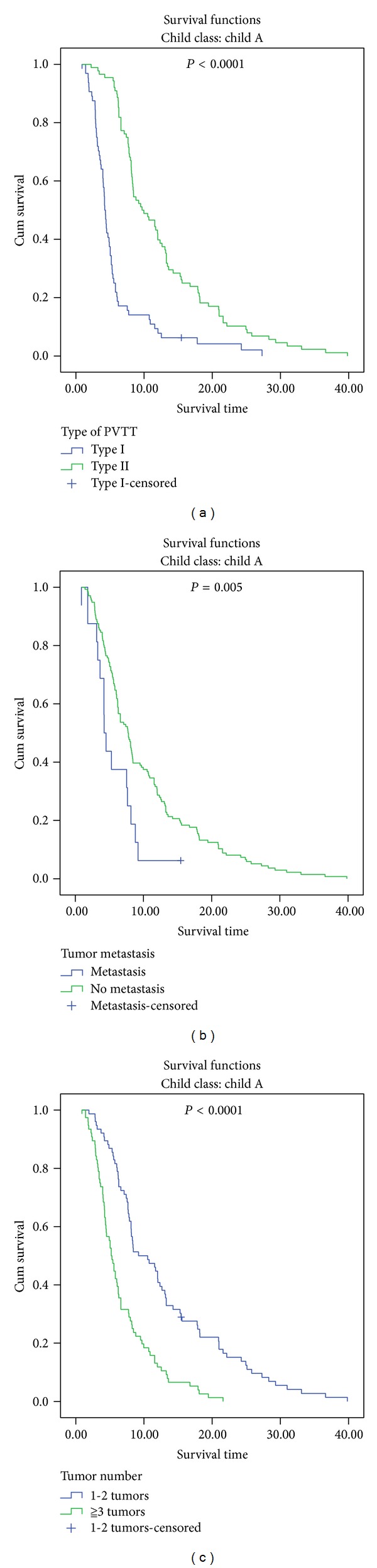
Subgroup comparison of overall survival in advanced hepatocellular carcinoma patients with portal vein tumor thrombosis and Child-Pugh class A according to the type of portal vein tumor thrombosis (a), metastasis (b), and number of tumor lesions (c).

The median overall survival was significantly longer in patients with Type II PVTT than in those with Type I PVTT (8.4 months versus 4.1 months, *P* < 0.0001) (Figure 1(b)). Among the Child-Pugh A group, the median overall survival of patients with Type I PVTT and those with Type II PVTT was 4.3 months and 9.8 months, respectively (*P* < 0.0001) (Figure 2(a)). For patients with Child-Pugh B, there was still significant difference between patients with Types I and II PVTT (3.4 months versus 5.6 months, *P* = 0.01).

The median overall survival was significantly longer in patients without extrahepatic metastasis than in those with (6.2 months versus 3.9 months, *P* = 0.0009) (Figure 1(c)). The significant difference remained in Child-Pugh A (extrahepatic metastasis: 4.4 months, versus no extrahepatic metastasis: 7.8 months, *P* = 0.01) (Figure 2(b)) and B (extrahepatic metastasis: 2.2 months, versus no extrahepatic metastasis: 4.3 months, *P* = 0.005) patients.

The median overall survival was significantly longer in patients with 1-2 tumor lesions than in those with ≧3 tumor lesions (8.1 months versus 4.5 months, *P* < 0.0001) (Figure 1(d)). The significant difference remained in Child-Pugh A patients (1-2 tumor lesions: 9.7 months, versus ≧3 tumor lesions: 5.3 months, *P* < 0.0001) (Figure 2(c)), but not in Child-Pugh class B patients (1-2 tumor lesions: 4.1 months, versus ≧3 tumor lesions: 3.7 months, *P* = 0.65).

### 3.3. Prognostic Factors

In univariate analysis, total bilirubin (HR = 1.012, 95% CI: 1.001–1.023, *P* = 0.026), Child-Pugh classification (HR = 4.324, 95% CI: 2.862–6.532, *P* < 0.001), type of PVTT (HR = 3.570, 95% CI: 2.623–4.851, *P* < 0.001),number of tumor lesions (HR = 2.589, 95% CI: 1.891–3.546, *P* < 0.001), and metastasis (HR = 2.206, 95% CI: 1.388–3.504, *P* = 0.0008) were associated with survival. In the multivariate analysis, Child-Pugh classification (HR = 2.981, 95% CI: 1.919–4.631, *P* < 0.001), type of PVTT (HR = 2.806, 95% CI: 2.024–3.890, *P* < 0.001), number of tumor lesions (HR = 2.288, 95% CI: 1.634–3.203, *P* < 0.001), and metastasis (HR = 1.909, 95% CI: 1.157–3.149, *P* = 0.011) were the independent predictors of survival (Table 2).

## 4. Discussion

The rationale of TACE was based on the fact that tumor growth mostly depends on the blood supply from hepatic artery in HCC patients [16]. Theoretically, this procedure could not be permitted in the presence of PVTT in HCC patients, because the coexistence of portal vein and hepatic artery obstruction potentially induces the development of liver failure and infarction [17]. In the current AASLD guidelines, the presence of PVTT is still considered as the main contraindication for TACE [4]. However, in Asia, many clinicians still consider TACE to be a useful treatment for patients with unresectable HCC and PVTT [18]. Two previous studies demonstrated that TACE could be safely performed in HCC patients with PVTT [11, 19]. Results from several comparative studies also supported the survival benefit of TACE in comparison with conservative treatment [12, 20–22]. The beneficial effect of TACE is further confirmed by a recent meta-analysis [13]. Nevertheless, because of the various inclusion criteria, the results of survival in these studies are quite heterogeneous ranging from 5 months to 8.7 months. Thus, it should be warranted to analyze the prognostic factors of advanced HCC patients with PVTT treated with TACE, thereby accurately selecting the candidates. Our study might be the biggest series ever reported in a single center to evaluate this issue. Two major findings of our study were as follows: (1) although a higher incidence of postembolization syndrome was observed, no procedure-related death occurred and (2) four baseline variables, including Child-Pugh classification, type of PVTT, number of tumor lesions, and metastasis, should be fully evaluated before TACE procedures in advanced HCC patients with PVTT.

Due to a high incidence of chronic hepatitis B virus infection, the incidence of HCC and its related death is higher in Asian countries than that in Western countries [23]. Indeed, TACE is often used for advanced HCC in Asian countries, such as China, Japan, and Korea [24]. Contrarily, the BCLC strategy and AASLD practice guidelines recommend that sorafenib is the sole standard treatment option of advanced HCC patients [3, 4]. A retrospective study by Pinter et al. showed that the median overall survival was 9.2 months (95% CI: 6.1–12.3 months) for 34 patients treated with conventional TACE with doxorubicin plus lipiodol or drug-eluting beads and 7.4 months (95% CI: 5.6–9.2 months) for 63 patients treated with sorafenib alone (*P* = 0.377) [9]. Their findings substantially challenged the current recommendation and supported the use of TACE in the setting of advanced HCC. Indeed, the median overall survival in our study (6.1 months) was comparable to that reported from one randomized controlled Asian trial in which advanced HCC patients were treated with sorafenib (6.5 months) [7]. Additionally, the complication rate of TACE in our study (88%) was comparable to that of sorafenib reported in the Asian trial (81.9%) [7]. More importantly, no death was attributed to the use of TACE, which was consistent with results from the use of sorafenib. Moreover, it should be noted that the cost of sorafenib is high and the economic level of HCC patients is often low in developing countries, thereby precluding the wide applications of this drug. Accordingly, the use of TACE in these patients should be considered.

In addition, although the incidence of procedure-related complications was 88%, these adverse events could be safely controlled and there was no procedure-related death. This important result demonstrated that TACE could be safely performed in patients with PVTT, even in cases with main portal vein obstruction. We consider that identification and superselective catheterization of tumor feeder vessels make a contribution to the safety of TACE procedure.

An important limitation of our study was the retrospective nature. But the data regarding survival status were accurate and well recorded by our team. Additionally, we did not include the patients with relatively poor liver function in our study. This was primarily because TACE might not be suitable for these patients and the survival benefit might be unclear.

## 5. Conclusions

This large retrospective study demonstrated that TACE could be selectively used for the treatment of advanced HCC with PVTT. But a high rate of postoperative adverse events should not be undermined in spite of no procedure-related death. Additionally, type of PVTT, number of tumor lesions, liver function, and metastasis are helpful for clinicians to predict the prognosis of these patients and select the candidates. Thus, HCC patients with Type I PVT, Child-Pugh B class, and extrahepatic metastasis might be considered poor candidates for TACE. Conversely, further prospective randomized controlled studies might be required to compare the efficacy of TACE with sorafenib in HCC patients with Type II PVT and Child-Pugh class A but without extrahepatic metastasis.

## Figures and Tables

**Table 1:** Patient baseline characteristics (*n* = 188).

Parameter	Number	%
Age/years, mean (range)	49.89 (18–80)	
Gender		
Male	167	88.3
Female	21	11.2
Ascites		
Yes	51	27.1
No	137	72.9
Tumor size/cm, mean (range)	8.8 (2–18.8)	
Number of tumor lesions		
≥3	102	54
<3	86	46
Extrahepatic metastasis		
Yes	22	11.7
No	166	88.3
Type of PVTT		
Type I	90	47.9
Type II	98	52.1
Arteriovenous Fistula		
Yes	32	17
No	156	83
Child-Pugh class		
A	152	80.9
B	36	19.1
ECOG		
0	18	9.6
1	168	89.4
2	2	1.1
Laboratory tests, mean (range)		
Alpha fetoprotein	40486 (1–121000)	
Hemoglobin (g/L)	130.1 (80–193)	
Platelets (10^9^/L)	146.8 (25–480)	
International normalized ratio	1.5 (0.83–38.3)	
Alanine aminotransferase (U/L)	53.7 (5–984)	
Aspartate aminotransferase (U/L)	83.8 (15–1242)	
Albumin (g/L)	37.8 (24–50.4)	
Total bilirubin (*μ*mol/L)	20.1 (6–112)	
Creatinine	80.2 (43–193)	

BCLC: Barcelona Clinic Liver Cancer; ECOG: Eastern Cooperative Oncology Group; AFP: *α*-fetoprotein; PVTT: portal vein tumor thrombosis.

**Table 2:** Predictors for survival after TACE in multivariate analysis.

Variables	Multivariate analysis		
	HR	95% CI	*P*
Total bilirubin	1.007	0.994–1.021	0.300
Child-Pugh class (class A versus class B)	2.981	1.919–4.631	<0.001
Type of PVTT (Type I versus Type II)	2.806	2.024–3.890	<0.001
Number of tumor lesions (≥3 versus <3)	2.288	1.634–3.203	<0.001
Extrahepatic metastasis (yes versus no)	1.909	1.157–3.149	0.011

TACE: transarterial chemoembolization; BCLC: Barcelona Clinic Liver Cancer; CI: confidence interval; HR: hazard ratio.

## Abbreviations

AASLD: American Association for the Study of Liver Disease
BCLC: Barcelona Clinic Liver Cancer
CI: Confidence interval
CT: Computed tomography
HCC: Hepatocellular carcinoma
HR: Hazard ratio
MPV: Main portal vein
PVTT: Portal vein tumor thrombosis
TACE: Transarterial chemoembolization.
